# Chemokines in progression, chemoresistance, diagnosis, and prognosis of colorectal cancer

**DOI:** 10.3389/fimmu.2022.724139

**Published:** 2022-07-22

**Authors:** Qian Zou, Xue Lei, Aijing Xu, Ziqi Li, Qinglian He, Xiujuan Huang, Guangxian Xu, Faqing Tian, Yuanlin Ding, Wei Zhu

**Affiliations:** ^1^ Department of Pathology, Guangdong Medical University, Dongguan, China; ^2^ Department of Genetics and Endocrinology, Guangzhou Women and Children’s Medical Center, Guangzhou Medical University, Guangzhou, China; ^3^ Department of Hematology, Longgang District People’s Hospital of Shenzhen, Shenzhen, China; ^4^ Guangdong Provincial Key Laboratory of Medical Molecular Diagnostics, School of Medical Technology, Institute of Clinical Laboratory, Guangdong Medical University, Dongguan, China; ^5^ School of Public Health, Guangdong Medical University, Dongguan, China

**Keywords:** chemokines, colorectal cancer, signal molecules, ncRNAs, immune escape

## Abstract

Plenty of factors affect the oncogenesis and progression of colorectal cancer in the tumor microenvironment, including various immune cells, stromal cells, cytokines, and other factors. Chemokine is a member of the cytokine superfamily. It is an indispensable component in the tumor microenvironment. Chemokines play an antitumor or pro-tumor role by recruitment or polarization of recruiting immune cells. Meanwhile, chemokines, as signal molecules, participate in the formation of a cross talk among signaling pathways and non-coding RNAs, which may be involved in promoting tumor progression. In addition, they also function in immune escape. Chemokines are related to drug resistance of tumor cells and may even provide reference for the diagnosis, therapy, and prognosis of patients with colorectal cancer.

## 1 Introduction

Colorectal cancer (CRC) is the third most common cancer and the second leading cause of death in all malignant tumors around the world (1). In 2018, there were over 1.8 million new cases of CRC patients, accounting for about 10.2% of all annually diagnosed cancers, and 881,000 deaths accounting for 9.2% (2). As cancer occurs, tumor cells will sustain a lasting interaction with the surrounding microenvironment. There are many factors that affect the development and immune response of tumors in this microenvironment, including various immune cells, stromal cells, tumor-derived exosomes, and cytokines (3–8). Chemokine, a small molecular weight secreted protein, is a member of the cytokine superfamily. Their main role in the tumor microenvironment (TME) is to induce the recruitment and migration of specific immune cells (9, 10). Chemokines are a kind of short peptides with low molecular weight (8–10 kDa) (11). They can be broken down into four main classes depending on the location of the first two cysteine (C) residues in their protein sequence, namely, CCL, CXCL, XCL, and CX3CL. Nearly all chemokines have four cysteines apart from XCL1 and XCL2 (9, 12). Directly or indirectly, chemokines influence tumor progression and chemoresistance and even have reference value for diagnosis and prognosis (12, 13). A large number of studies and attempts have been made to exploit the features of chemokines. However, due to the multidirectional property and pleiotropy of regulatory networks, how to apply the research to the clinic remains a challenge.

In this review, we expound the important functions of chemokines in the tumor microenvironment from five aspects: attractors of immune cells, signaling molecules, regulation of non-coding RNAs (ncRNAs), tumor immune escape, and extrinsic factors. We also analyze and summarize the role in chemoresistance as well as diagnosis and prognosis.

## 2 Chemokines and tumor progression

### 2.1 Chemokines involved in tumor progression as attractors of immune cells

Chemokines are initially regarded as an inflammatory factor involved in acute inflammation (10). They recruit immune cells to the injured sites and affect tumor invasion or migration by assembling the infiltration of immune cells into tumor tissue. Bioinformatics analyses about the relationship between the 12-chemokine (CCL2 (chemokine C–C motif ligand 2), -3, -4, -5, -8, -18, -19, -21, CXCL9 (chemokine C–X–C motif ligand 9), -10, -11, -13) signature and immune inflammation have identified that high signature significantly linked to more tumor-infiltrating immune cells, such as cytotoxic T lymphocytes, B cells, monocytes, and myeloid dendritic cells (DCs) (14). These stereotypical chemokines are discussed below, along with recent insights into chemokines control of immune cells in CRC.

#### 2.1.1 T lymphocytes

As a protector in organisms, high density of activated CD8^+^ T lymphocytes infiltrates in tumor sites in connection with better prognosis in patients with CRC. However, activated CD8^+^ T lymphocytes are significantly reduced in patients with the advanced stage of cancer (15, 16). CD8^+^ T cells express CXCR3 on their surface; the ligands of CXCR3 are CXCL9, CXCL10, and CXCL11. Therefore, CD8^+^ T cells are recruited into the site of the tumor in response to those chemokines and play an efficient role against cancer (12, 17–19). For example, tumor necrosis factor superfamily member 4, an immunostimulatory factor, elicits the production of CXCL9 by mesenchymal stem cells (MSCs) which exert the antitumor function relying on recruitment of CD8^+^ T cells (20). The expression of CXCL10 in tumor blood vessel endothelial cells is increased by Treg depletion, leading to further promotion of T-cell migration into tumors (21). Additionally, IL-17 signals can be acted on CRC cells and decrease the production of CXCL9/10. Subsequently, the infiltration of CD8^+^ cytotoxic T lymphocytes and Tregs to CRC is inhibited, thus promoting CRC development (22). However, CXCL11 seems to be opposite from the original expectations. CXCL11 is up-regulated in CRC cell lines and tissues. Restraining the CXCL11 expression significantly inhibits epithelial–mesenchymal transition (EMT) and the migration and invasion of CRC cells (23).

#### 2.1.2 Tumor-associated macrophages

Macrophages with different polarizations coexist in tumor tissues. In response to various signals *in vivo*, they can be activated and differentiate into M1 and M2 macrophages (24). The M1 macrophages release proinflammatory mediators and facilitate tumoricidal activity. Conversely, the M2 phenotype is considered to be involved in tumor progression and to have immunosuppressive functions (25, 26). Moreover, the M1 phenotype is correlated positively with survival, while enrichment of the M2 phenotype is correlated with cancer death (27). Tumor-associated macrophages (TAMs) have been reported to be associated consisting of highly expressed CCL2, CCL3, CCL5, CCL18, CXCL1, and CXCL12 in the TME (28). The homeoprotein Six1, a member of the Six family of homeodomain transcription factors, increases the expression of CCL2, CCL5, and vascular endothelial growth factor (VEGF), attracts macrophage infiltration into the tumor, and further leads to CRC tumor growth, progression, and metastasis (29). Deletion of CCR6 (CCL20 receptor) decreases macrophage infiltration and is associated with reduced tumor burden in diminished formation of intestinal adenoma formation in a model of sporadic intestinal carcinogenesis (APC^MIN/+^ mouse model) (30).

Furthermore, chemokines not only are attractants for leukocytes but also induce a different differentiation program in monocytes. For instance, the secretion of CCL2 induced by NOD-like receptor protein 7 activates the NF-κB signaling pathway and switches macrophages to an M2 phenotype in CRC (31). Depending on the CCL18 secretion in tumor-decellularized matrices, macrophages differentiate toward an anti-inflammatory phenotype and then facilitate CRC cell invasion (32). There is another mode of shifting phenotype that also contributes to this goal. Exosomes from CXCR4-overexpressing CRC cells deliver certain miRNAs to macrophages causing the polarization of pro-tumoral M2 macrophages through activating the PTEN/PI3K/Akt signaling pathway. In turn, the M2 macrophages reinforce angiogenesis and liver metastasis of CRC by secreting VEGF and promoting EMT (33). Cancer-associated fibroblasts (CAFs) attract monocytes by secreting CXCL8 (IL-8) and subsequently contribute to M2 polarizations, which synergize with CAFs in suppressing the functioning of NK cells in CRC (34). Hence, this is one way to be involved in tumor development by affecting the polarization of immune cells.

#### 2.1.3 Tumor-associated neutrophils

Neutrophils are differentiated from a myelocytic lineage. CXCR2 binds to its ligands, including CXCL1, CXCL2, CXCL3, CXCL5, CXCL7, and CXCL8, in charge of the recruitment of neutrophils (35–37). Like macrophages, neutrophils play different roles in tumor immune response because of the development of differentiated phenotype. Some studies pointed out the tumor-cytotoxicity effect of neutrophils; nevertheless, some other studies suggested that neutrophils have been implicated in promoting the metastasis of tumor cells. In addition, neutrophil numbers and neutrophil-related factors have something to do with cancer progress (38–40).

**Figure 1:**
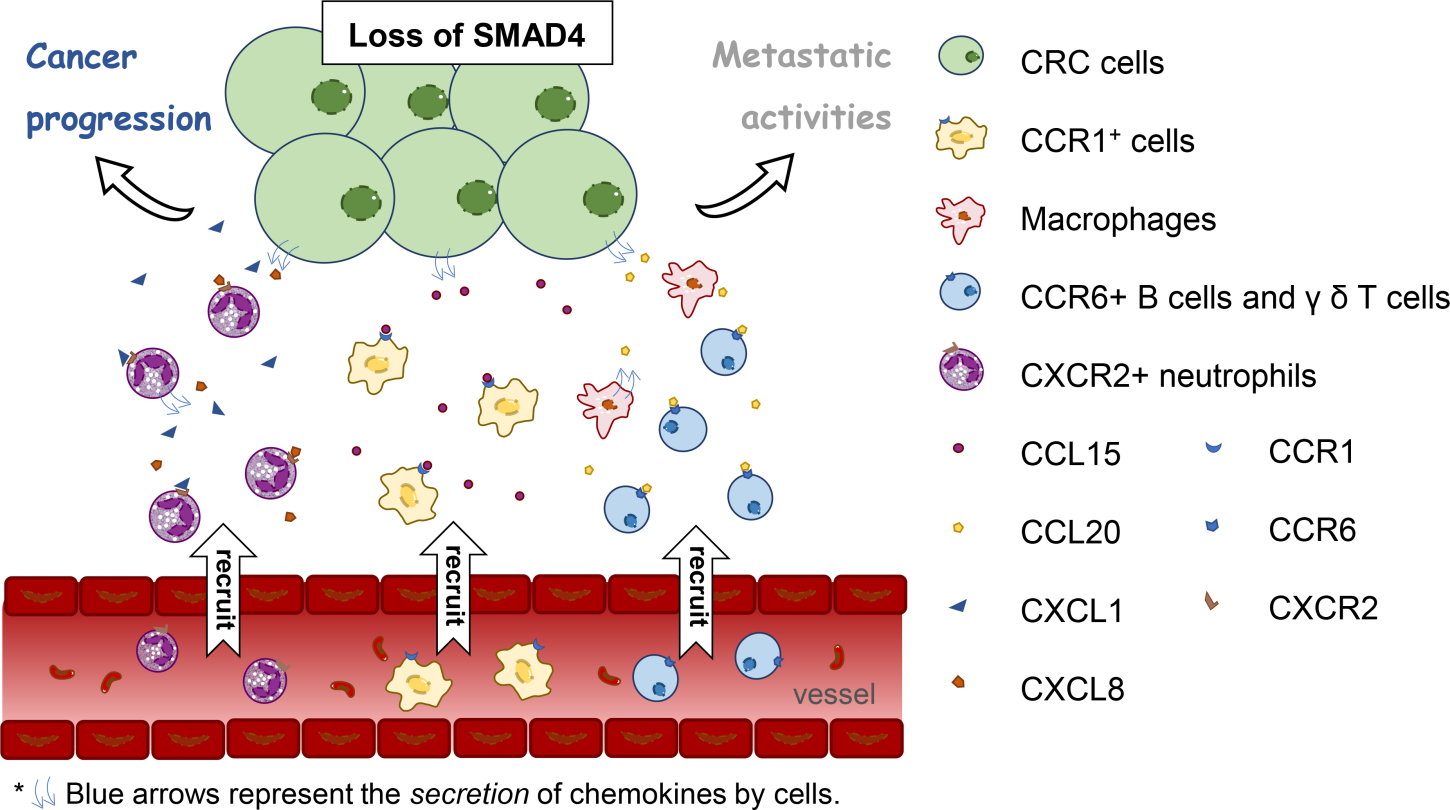
The relation between loss of Mothers Against Decapentaplegic Homolog 4 (SMAD4) in human colorectal cancer cells and tumor-infiltrating immune cells and its chemokines and receptors.

The colonic epithelial hypoxic response is involved in colonic tumorigenesis in mouse models of colitis-associated cancer (CAC) by activating HIF-2α, which mediates neutrophil recruitment *via* the CXCL1–CXCR2 axis (41). Another transcription factor, Mothers Against Decapentaplegic Homolog 4 (SMAD4), concatenates immune cells and tumor by chemokines. SMAD4 is a key transcription factor of TGF-β signaling and a tumor suppressor. Knockdown of SMAD4 from human CRC cells enhances CXCL1 and CXCL8 expression and accumulates CXCR2^+^ neutrophils to CRC tumor. Subsequently, those neutrophils produce more CXCL1 and CXCL8 and then appeal to CXCR2^+^ neutrophils. This forms an amplification of the cytokine/chemokine milieu in CRC (42). Loss of SMAD4 causes CCL15 secretion which helps in the accumulation of CCR1^+^ cells at the invasion front of primary CRC and promotes their metastatic activities to the lung (43). In addition, SMAD4 depletion is associated with up-regulation of CCL20 (44). CCL20 is in charge of recruiting lymphoid cells and DCs and macrophage infiltration (30). Meanwhile, CCL20 produced by tumor-promoting macrophages promotes CAC progression by recruiting CCR6^+^ B cells and γ δ T cells (45). Thus, the defective SMAD4 in human CRC cells may seem to be correlated with tumor-infiltrating immune cells and its chemokines and receptors. A new direction is provided for the relationship between infiltration of immune cells and CRC (**Figure 1**). However, as a consequence of the difference between mouse models for basic research and humans in tumor evolution and immune behavior, this may affect diverse reactions of chemokines (46). Thus, of additional concern when translating these findings to the clinical arena is the inherent distinction between mice and humans in the biological characteristics.

#### 2.1.4 Endothelial cells

The survival and metastasis of solid tumors cannot be separated from angiogenesis processes. Endothelial cells as the interface between blood and surrounding tissues play a critical role in inflammation and angiogenesis (47). Chemokines are one of the main tools for endothelial cells. Endothelial cells are activated by reciprocal action or dynamic change of tumor cells and leukocytes and then produce certain chemokines. A straightforward example is that Treg depletion leads to the elevated production of CXCL10 from endothelial cells in the tumors and facilitates T-cell migration into tumors (21). Chemokines bind to specific transmembrane receptors and subsequently activate or inhibit intracellular signaling pathways like VEGF signaling pathway. CXCL5 combines with CXCR2 to promote angiogenesis through the activation of the AKT/NF-κB/FOXD1/VEGF-A pathway (48). Additionally, CXCL1, CXCL2, CXCL4, CXCL7, CXCL8, CXCL11, CXCL12, and some chemokine receptors are also associated with angiogenesis (49–53). For instance, CCR6 is found significantly overexpressed in CRC tissues and facilitates tumor angiogenesis *via* the AKT/NF-κB/VEGF pathway (54). However, CCL19 high expression inhibits CRC angiogenesis by inhibiting the Met/ERK/Elk-1/HIF-1α/VEGF-A pathway (55). Surely VEGF, in turn, also regulates the expression of chemokines, thus affecting angiogenesis and CRC development. 5′-Nucleotidase domain-containing 2 (NT5DC2) is significantly upregulated in CRC tissues and cell lines. Its inhibition reduces the activation of CCL2/CCR2 and AKT/NF-KB signaling pathways as well as angiogenesis and TAM infiltration in CRC cells. These effects mainly depend on regulation by NT5DC2 of VEGF (56). Another example about the link between VEGF and CCL2 is that the concurrent use of anti-VEGFA (bevacizumab) and anti-CCL2 therapy inhibits tumor angiogenesis and growth more effectively than treatments alone in CRCs with ETV5 high expression (57). E26 transformation-specific variant 5 (ETV5)-mediated angiogenesis is dependent on the VEGFA pathway in CRC (58). As these examples illustrate, chemokines and VEGF activate or restrain each other and influence tumor angiogenesis and metastasis.

#### 2.1.5 Cancer-associated fibroblasts

Fibroblasts are the main cellular components of TME and derived from MSCs (59). Those activated fibroblasts in the TME are called CAFs. Activated fibroblasts can provide cancer cells with scaffolds and growth factors to promote CRC cell invasion, metastasis, and chemoresistance (60–62). Fibroblasts are a source of chemokine production. Moreover, their recruitment and differentiation are also regulated by chemokines or chemokine receptor pathway. Researchers found that fibroblasts secrete CXCL5, CXCL12, CCL1, and CCL2 (61–64). IL-6 and CXCL8 released from myofibroblasts stimulate myeloid cells which differentiate into S100A8/9-expressing MDSCs or M2 macrophages in the CRC microenvironment (65). In recent years, investigators have also found that CAFs produce CXCL8 and then contribute to M2 polarizations (34). Recruitment of the fibroblasts is controlled by increased CXCL1, CXCL8, and CCL3 expression in tumors (66, 67). In *in vivo* xenograft experiments, MSCs promote the differentiation into CAFs through CXCR4/TGF-β1 signaling in either primary tumor tissues or hepatic metastatic tissues of CRC (68). Taken together, CAFs act as important cell components in the TME, produce chemokines, or are activated by chemokines and further affect the recruitment and differentiation of their own or other cells. These events cause further tumor development and chemoresistance.

### 2.2 Chemokines mediate tumor immune escape

**Figure 2:**
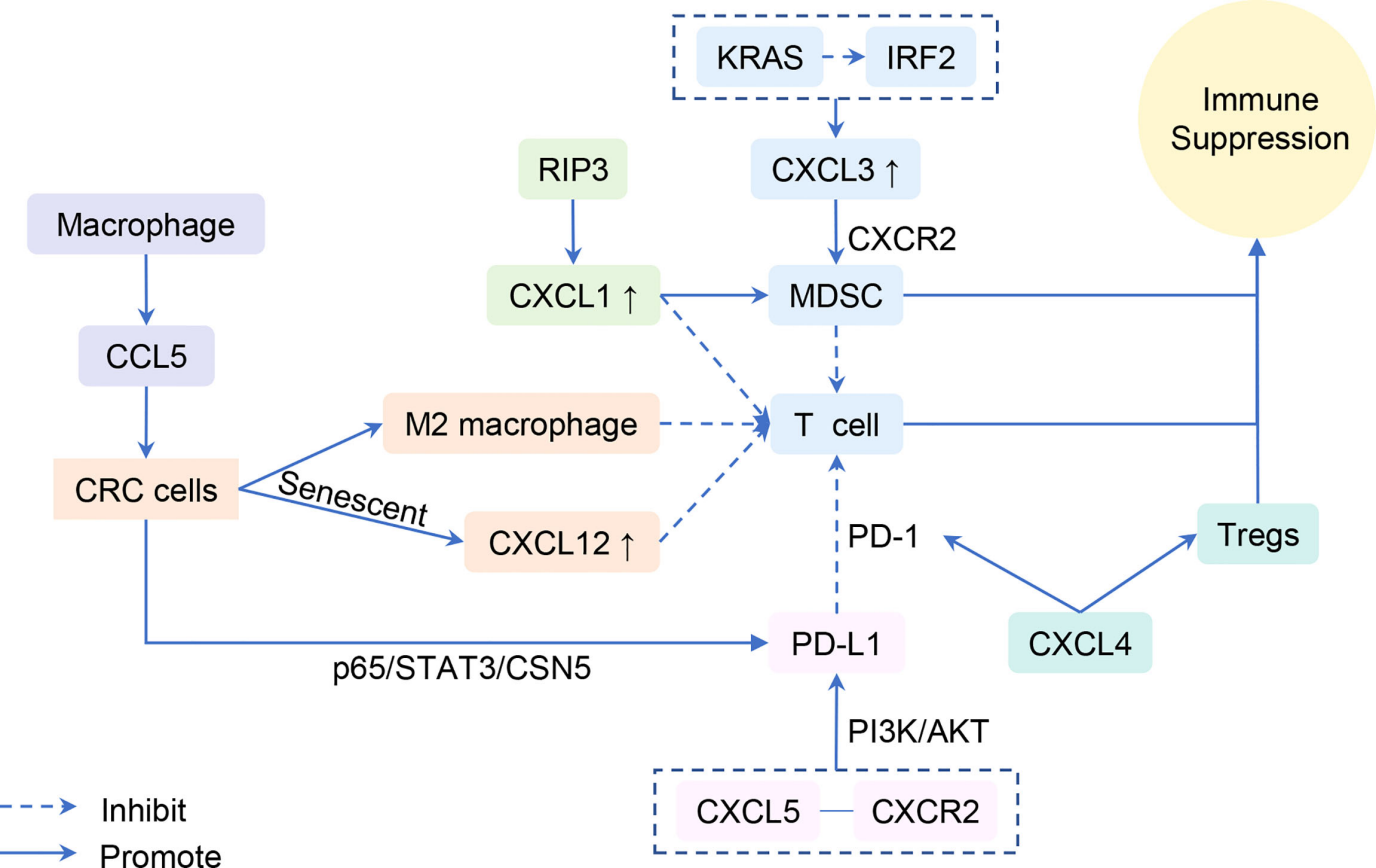
Chemokines are involved in the immune evasion.

Immune escape of tumor cells involves multiple steps, links, and factors. Immune cells, including negative immune regulation by MDSCs and defective antitumor T cells, are directly involved in the antitumor immunological effect of the body and tumoral immune escape (69–71). Consequently, chemokines play an important role in the immune evasion owing to the countless connections between immune and chemokines (**Figure 2**).

The secretion of CXCL1 is up-regulated in the CAC model by overexpression of receptor-interacting protein 3, which mediates a regulated necrotic cell death modality in a caspase-independent fashion. Additionally, CXCL1-induced adaptive immune suppression leads to CAC progression by promoting the recruitment of MDSCs and M2-like macrophage but inhibiting infiltration and activation of T cells (72). Similarly, the KRAS-induced immunosuppressive tumor microenvironment evades the T-cell responses through the IRF2-CXCL3-CXCR2 pathway and immunosuppressive myeloid cells. Inhibition of this axis profoundly impairs tumor growth and progression (73). Moreover, the combination of CXCL5 and CXCR2 elevates programmed death-ligand 1 (PD-L1) expression in mouse tumor cells, depending on the activation of PI3K/AKT signaling (63). PD-L1, a membrane-spanning protein, binds to its inhibitory receptor programmed death 1 (PD-1) on T cells, causing escape and obstructing the activation of T lymphocytes (74, 75). CXCL4 was also found to influence the activation of CD8^+^ T cells and then affect cancer growth. Non-platelet-derived CXCL4 exhibits an immunosuppressive role and accelerates CRC growth. Mechanistically, CXCL4 enhances PD-1 expression and apoptosis of CD8^+^ T cells and inhibits CD8^+^ T-cell activation yet promotes Treg proliferation and reduces PD-1 expression (76). We speculate that CXCL4 is involved in T-cell-mediated immune escape.

CXCL9 and CXCL10 produced by myeloid cells in CRC liver metastases induce tumor-infiltrating lymphocytes to invade the margins. Those lymphocytes secrete CCL5, which leads to tumor growth and invasion (77). However, contrary to what was mentioned in the part of T lymphocytes, CXCL9 and CXCL10 here induce tumor invasion indirectly. In another study, researchers explored and supplemented that CCL5 secreted by macrophages inhibit the CD8^+^ T-cell-mediated killing behavior of HT29 cells and promote immune escape by activating the p65/STAT3/CSN5 signaling axis to deubiquitinate and stabilize PD-L1, which may form a positive feedback loop and boost the infiltration of macrophages, subsequently promoting the growth of CRC (78). Therefore, knockdown of CCL5 facilitates CD8^+^ T-cell infiltrate into the central tumor area and delays tumor growth and metastasis (79).

Finally, aging tumor cells may form barriers of chemokines that protect the other tumor cells from attacking the immune system. Senescent CRC cells inhibit the infiltration of CD8^+^ T cells by inducing a high concentration of CXCL12. As such, this elicited the loss of CXCR4 on T cells and caused the ruination of directional migration (80).

In conclusion, immune evasion and cancer progression were regulated by the expression of chemokines, chemokine receptors, and PD-1/PD-L1, as well as associated signaling pathways and immune cells.

### 2.3 Chemokines activate certain signaling pathways as signaling molecules

**Figure 3:**
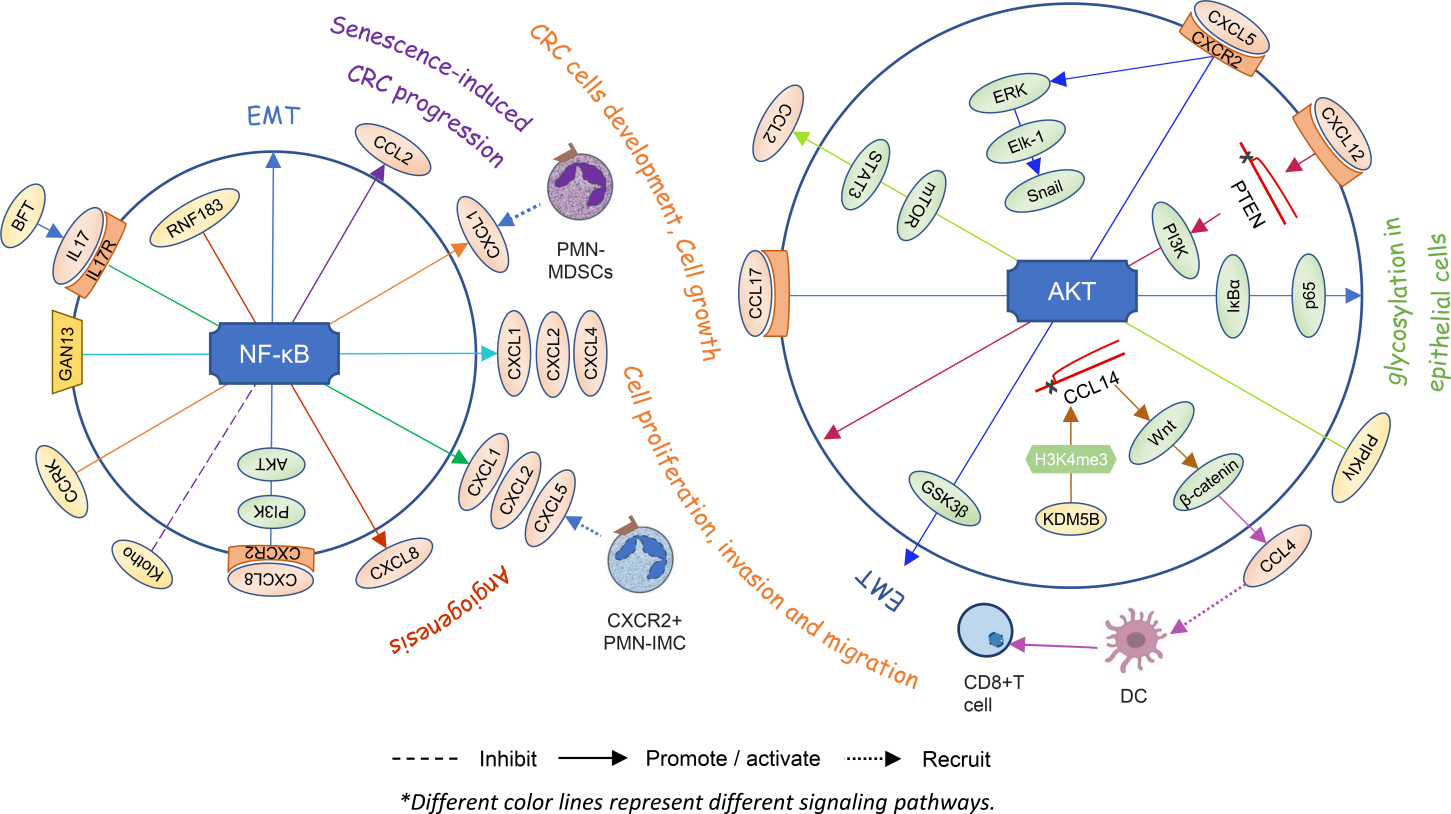
Chemokines and signaling pathways within cells directly affect each other’s states and the angiogenesis or proliferation and invasion of colorectal cancer.

Chemokines are one of the important components in the tumor microenvironment. As a signal factor, they participate in various intracellular signaling pathways and play a corresponding role in tumor cells and the certain cells (**Figure 3**).

#### 2.3.1 NF-κB signaling pathway

Utilizing a colonic tumor model of Apc^Min^ mice induced by enterotoxigenic Bacteroides fragilis (ETBF), researchers found that the bacteroides fragilis toxin (BFT) from ETBF selectively activates NF-κB signaling in the distal colon, depending on IL-17 and IL-17R in colonic epithelial cells. Then, a proximal to distal mucosal gradient of chemokines is formed, including CXCL1, CXCL2, and CXCL5, which attracts CXCR2^+^ polymorphonuclear immature myeloid cells (PMN-IMC) and eventually induces distal colon tumorigenesis (81). GNA13, belonging to a subfamily of G protein-coupled receptors of the Gα subunit, promotes tumor growth and angiogenesis by upregulating CXCL1, CXCL2, and CXCL4 *via* the IKKβ/NF-κB signaling pathway in CRC cells (49). Hepatocyte-intrinsic cell cycle-related kinase (CCRK) also upregulates CXCL1 production depending on the NF-κB signaling. Elevated CXCL1 leads to enrichment of polymorphonuclear myeloid-derived suppressor cells (PMN-MDSCs) in the liver microenvironment and disturbs NK cell-mediated immunosurveillance, thereby creating a pre-metastatic niche for liver metastasis (82). Overexpression of CXCL8 induces EMT of LoVo cells and promotes cell proliferation, migration, and invasion by activating the PI3K/AKT/NF-κB signaling axis (83). The RING finger gene 183 (RNF183), a CRC-associated gene, upregulates the expression of chemokine CXCL8 by the NF-κB pathway and promotes cancer cell growth, migration, invasion, and metastasis *in vitro* and *in vivo* (84). It is speculated that there may be positive feedback promoting the progression of CRC *in vivo*.

Cellular senescence is considered to be a protective mechanism against tumorigenesis in response to excessive extracellular or intracellular stress. However, recent reports have suggested that senescent stromal cells promote CRC cell progression by secreting CCL2. Klotho protein is a classical antiaging protein. The CCL2 expression-related NF-κB signaling pathway is weakened when HEK293 cells are pretreated with Klotho (85). In other words, the NF-κB/CCL2 signaling pathway might be involved in senescence-induced CRC progression.

#### 2.3.2 AKT and β-catenin signaling pathway

Expression of CXCL5 produced by cancer epithelial cells promotes the migration and invasion of CRC cells. CXCL5 activates the AKT/GSK3β/β-catenin pathway and ERK/Elk-1/Snail pathway in a CXCR2-dependent manner and then induces the EMT (86). Lysine demethylase 5B is a member of the histone lysine demethylases which inhibit CCL14 transcription *via* demethylation of H3K4me3, ultimately activating the Wnt/β-catenin pathway and leading to the tumorigenesis of CRC in cells and animal models (87). Meanwhile, β-catenin also has an impact on the expression of chemokines. It regulates the production of CCL4, which recruits CD103^+^ DCs and finally activates CD8^+^ T cells in the tumor area (88). High-expression PIPKIγ stimulates the AKT-mTOR signaling activation, leading to increased STAT3 phosphorylation and ultimately promoting CCL2 expression which further facilitates macrophage infiltration and suppresses the activation of CRC tumor immune response (89). The production of CCL17 produced by M2 macrophage cocultures with colon cells activates IκBα and p65 *via* the AKT pathway that leads to glycosylation in epithelial cells and promotes the development of ulcerative colitis and colitis-associated CRC (90). Stromal cell-derived CXCL12 down-regulates PTEN and activates the PI3K/Akt pathway, resulting in proliferation and invasion in CRC cells (91). Intestinal microbiota has an inseparable relation with carcinogenesis. DCs increase CXCL13 expression when it comes to intestinal microbiota translocation. CXCL13 recognizes and binds to its receptor CXCR5 on the epithelial cell surface and then activates the AKT signaling pathway and the consequent CAC tumorigenesis (92).

It follows then that chemokines and signaling pathways within cells directly affect the each other’s states and the angiogenesis or proliferation and invasion of CRC.

### 2.4 Chemokines and ncRNAs engender a bidirectional effect

Plenty of studies have proved that ncRNAs play a key role in either normal cellular function or various diseases, including various cancers. NcRNAs account for nearly 90% of the RNAs made from the huge human genome. Among the common classes are microRNAs (miRNAs), circular RNAs (circRNAs), and long ncRNAs (lncRNAs) (93). These RNAs are not involved in producing proteins but naturally link to gene networks and the regulation of signaling pathways (94). Not surprisingly, there seems to be an interplay between chemokines and ncRNAs (95, 96).

On the one hand, ncRNA regulates the expression of chemokines in the CRC tumor cell and performs its recruitment or antitumor functions (**Table 1**). For instance, cricGLIS2 (has_circRNA_101692)-overexpressing cells induce the expression of CXCL1 and CXCL8 in an autocrine and paracrine manner and enrich neutrophils (97). circCTNNA1 is up-regulated in CRC tissues and cells, which sponges miR-363-3p and curbs CRC progression by regulating CXCL5 expression (98). miR−432−5p also targets CXCL5 and functions as a tumor suppressor to inhibit the migration and invasion of CRC cells (99). miR-204 is down-regulated in tumor compared with adjacent normal colon tissues (100). It is regarded as a tumor-suppressive miRNA in CRC cell lines (101). miR-204 represses the expression of CXCL8 by regulating the PI3K/AKT/mTOR signaling pathway. It exerts antitumor functions by curbing the viability, migration, and invasion of CRC cells, increases the apoptotic cell rates, and participates in inhibition of EMT in CRC cells (102). Exosomal−miR−10a produced by CRC cells reduces CXCL8, IL−6, and IL−1β secretion and refrains from the proliferation and migration in primary normal human lung fibroblasts (103). This prompts that miR−10a may be engaged with the process of CRC lung metastasis by changing the immune microenvironment around primary cells. miR15A and miR16-1 decrease CXCL9 and CXCL10 secreted by epithelial cells *via* NF-κB and STAT1 pathways and inhibit IgA^+^ B-cell infiltration. These IgA^+^ B cells inhibit the proliferation and activation of CD8^+^ T cells and exert immunosuppression (104). The deletion of miR-183-5p down-regulates levels of PD-L1, CCL1, CCL4, and CCL7 and immune-related proteins (EGFR, STARD1, and STARD3) and promotes apoptosis and inhibits proliferation of HCT116 cells (106). Small nucleolar RNA host gene 17 (SNHG17), belonging to lncRNAs, is highly expressed in colorectal adenocarcinoma (CRA) cells. It promotes tumor cell proliferation and migration on CRA cells by sponging miR-23a-3p to up-regulate CXCL12 (105). A novel lncRNA u50535, greatly overexpressed in CRC tissues, regulates CCL20 expression *via* activating its promoter and affecting CCL20/CCR6/ERK signaling and ultimately leads to cell proliferation and migration in CRC (107).

**Table 1:** ncRNA regulates the expression of chemokines in the CRC tumor cell and perform its recruitment or antitumor functions.

NcRNAs	Regulation or signaling pathway	Target chemokines	Reference
cricGLIS2	/	CXCL1, CXCL8 ↑	(97)
circCTNNA1	Sponges miR-363-3p	CXCL5	(98)
miR−432−5p	/	CXCL5	(99)
miR-204, miR−10a	PI3K/AKT/mTOR	CXCL8 ↓	(100–103)
miR15A, miR16-1	NF-κB and STAT1	CXCL9, CXCL10↓	(104)
SNHG17	Sponges miR-23a-3p	CXCL12 ↑	(105)
miR-183-5p	/	CCL1, CCL4, CCL7	(106)
lncRNA u50535	Activates its promoter	CCL20 ↑	(107)

On the other hand, chemokines, in turn, affect the gene regulation of ncRNAs and further embroil themselves in tumor progression (**Table 2**). The up-regulation of lncRNA X-inactive-specific transcript (XIST) induced by CXCL12/CXCR4 mediates sponge miR-133a-3p, thereby facilitating inflammatory CRC progression and invasion through recruiting macrophages and up-regulation of RhoA/ROCK signaling (108). In addition, there are other miRNAs, including miR-25-3p, miR-130b-3p, and miR-425-5p, as mentioned above, that are up-regulated in CRC cells by activation of the CXCL12/CXCR4 axis (33). CXCL1 derived from tumor-associated DCs increases cell mobility, cancer migration, and EMT of CRC by increasing miR-105 (95). A low expression of CCL19 in CRC is related to tumor angiogenesis, and a high expression of CCL19 inhibits CRC angiogenesis by promoting miR-206, thus inhibiting the Met/ERK/Elk-1/HIF-1α/VEGF-A pathway (55). Co-stimulation of tumor cells with CCL20 and CXCL8 decreases circ_0026344 expression and facilitates the proliferation, metastasis, and EMT of CRC (109). Even a possible relationship between chemokines and ncRNAs may be cyclical: miR125b is overexpressed by stimulating the CXCL12/CXCR4 axis in CRC, which in turn up-regulates the expression of CXCR4. This positive feedback loop activates the Wnt/β-catenin signaling by targeting adenomatous polyposis coli gene and subsequently promotes EMT and cancer invasion (96). The expression of chemokines and ncRNAs is linked, and the interactions go both ways.

**Table 2:** Chemokines affect the gene regulation of ncRNAs and further embroil itself in tumor progression.

Chemokines	NcRNAs	Regulation	Reference
CXCL12/CXCR4	lncRNA XIST, miR-133a-3p	(+)RhoA/ROCK signaling	(108)
CXCL12/CXCR4	miR125b, miR-25-3p,	/	(33, 96)
	miR-130b-3p, miR-425-5p	/	
CXCL1	increasing miR-105	EMT	(95)
CCL20、CXCL8	circ_0026344 ↓	EMT	(109)
CCL19	miR-206	(-) Met/ERK/Elk-1/HIF-1α/VEGF-A	(55)

### 2.5 Chemokines act as bridges between some extrinsic factors and tumor

**Figure 4:**
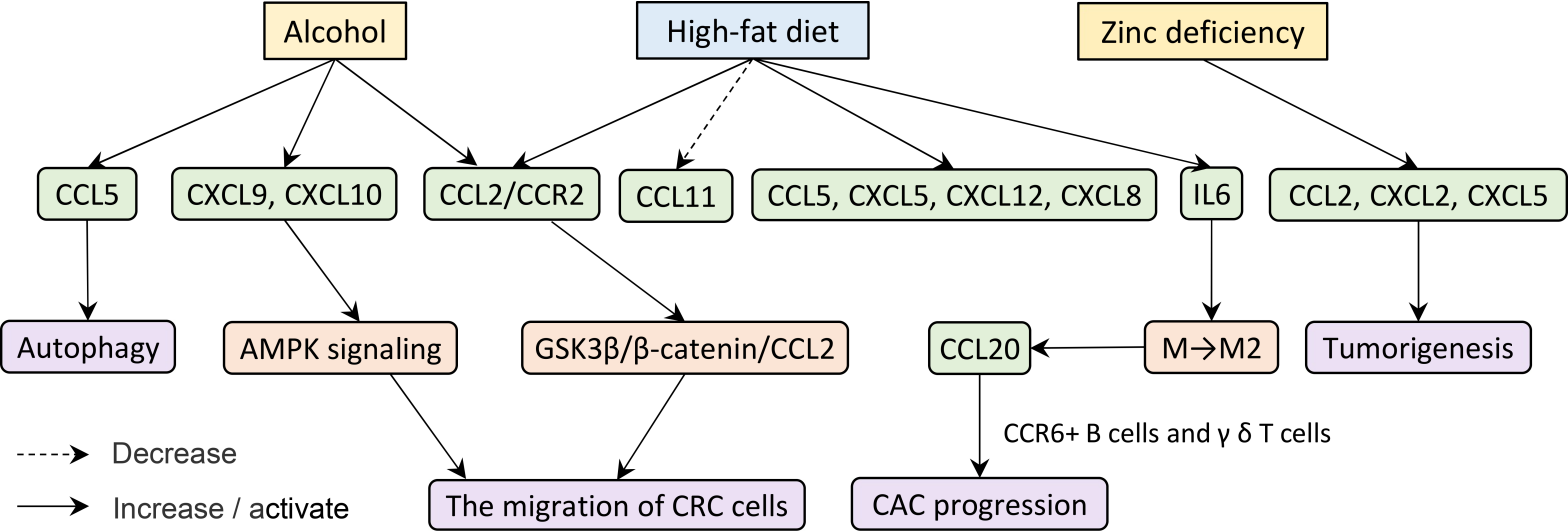
Excessive alcohol consumption and obesity have an effect on tumorigenesis and development.

Already it is known that tumorigenesis and development are not only susceptible to their nature but also affected by extraneous factors such as smoking, excessive alcohol consumption, and obesity (110–112). Here we find some key clues in diet, such as excessive alcohol intake, high-fat diet (HFD), and deficiency of trace elements (**Figure 4**).

A study in mice demonstrated that chronic ethanol feeding significantly increases the expression of CCL5, CXCL9, and CXCL10 in the colonic mucosa of azoxymethane/dextran sodium sulfate (AOM/DSS)-treated mice (113). Alcohol elicits CCL5 expression which in turn induces autophagy and increases the migration of CRC cells *via* activating AMPK signaling (114). Furthermore, CRC cells up-regulate CCL2/CCR2 in responding to alcohol exposure. Ultimately, alcohol promotes the metastasis of CRC through modulating the GSK3β/β-catenin/CCL2 pathway (115). The expression of CCL2/CCR2 in CRC patients with HFD is also significantly higher than that in CRC patients with a normal diet (116–118).

The chemokine protein array shows elevated plasma concentrations of CXCL5 and CXCL12 in the HFD group (119). Not only that, the expression levels of IL-6, CXCL8, and CCL2 secreted by adipocytes from obese and CRC subjects are significantly higher compared to normal-weight subjects (120). Another study found that obesity-induced IL-6 shifts macrophage polarization toward tumor-promoting macrophages, namely, M2 type, which produces CCL-20 in the CAC microenvironment. As noted earlier, CCL-20 promotes CAC progression by recruiting CCR6^+^ B cells and γ δ T cells (45). Moreover, low-density lipoprotein cholesterol upgrades CCL5 but reduces CCL11 expression in the CRC (121). As mentioned before, many studies considered that CCL5 promotes tumor migration, invasion, and even immune escape (29, 77, 78).

Apart from that, certain trace element deficiencies may cause the cancer to grow. Zinc deficiency, for example, significantly promotes the mRNA and protein levels of CCL2, CXCL2, and CXCL5 in the colonic mucosa and tumorigenesis in the colon (122).

## 3 Chemokines and chemoresistance

Over the past few decades, significant progress has been made in the development and use of an effective therapeutic method for CRC, including surgery, radiotherapy, chemotherapy drug, molecularly targeted therapy, and biological immunotherapy (123). Many effective drugs for the treatment of CRC have led to substantial improvement of overall survival; however, the clinical effects are not ideal due to the development of resistance (124). There are several mechanisms responsible for drug resistance including drug efflux, epigenetic modifications, environmental factor epigenetics, or the formation and survival of drug-tolerant cells (125). Here, we find that chemokines facilitate the occurrence of drug resistance through various signaling pathways or by increasing stem cell properties.

### 3.1 5-fluorouracil

Among various drug treatments, 5-fluorouracil (5-FU), a widely employed anti-metabolite, is the foundation of standard chemotherapy for CRC (126). 5-FU belongs to an analog of uracil, and its C5 position is replaced by a fluorine substitution (127). Therefore, it mainly acts as a thymidylate synthase inhibitor that blocks thymidylate synthase to inhibit DNA formation and induce lack of normal RNA. This consequence is an unbalanced cell growth that leads to the demise of those rapid-growing tumor cells due to a large accumulation of lethal 5-FU (128). However, CRC cells can survive by inhibiting 5-FU-induced cell death *via* modulation of apoptosis, autophagy, cell cycle, glucose metabolism, oxidative stress, mitochondrial activity, EMT, and so on (129). Chemokines act as a link in various signaling pathways by binding with their specific receptors.

Clinical studies showed that CCL20, CXCL13, and CXCR5 levels are significantly higher in the serum 5-FU-resistant CRC patients (130, 131). CCL20 secreted by CRC cells induces the recruitment of Tregs and promotes chemoresistance of CRC cells to 5-FU *via* FOXO1/CEBPB/NF-κB signaling (131). As for the elevation of CXCL13 in the patients with 5-FU resistance, it is possible that additional elevation is caused by 5-FU or that the 5-FU resistance is induced by CXCL13 and CXCR5 secretion. One study found that CXCL13 released by M2 polarization of macrophages activates the CXCL13/CXCR5 axis in tumor cells and promotes CRC liver metastasis. Here, the reason for secretion of CXCL13 is that exosome miR-934 derived by CRC cells induces M2 polarization in macrophages *via* down-regulation of PTEN expression and activation of the PI3K/AKT signaling pathway. Moreover, even positive feedback may also exist (132). However, it remains unclear whether this pathway participates in 5-FU resistance. However, it is certain that CXCL13 causes 5-FU chemoresistance (130). In addition, CCL21 significantly increases the IC50 of doxorubicin and 5-FU as well as mammosphere forming ability with up-regulation of P-glycoprotein (P-gp) and stem cell properties. Snail plays a vital role in CCL21 promoted chemoresistance and cancer stem cell properties (133). Moreover, CCL1 secreted by Snail-expression fibroblasts may also participate in anti-apoptosis, pro-proliferation, and 5-FU resistance through TGF-β/NF-κB signaling pathways in mouse models (62). In consequence, the investigators speculated that snail may serve as a predictive biomarker of the cellular response to 5-FU and a therapeutic target for 5-FU-based chemotherapy.

### 3.2 Anti-PD-1 or PD-L1

Tumor cells escape host antitumor immune response, mentioned above, through the PD-1/PD-L1 interaction in the tumor microenvironment. The immune checkpoint inhibitors (ICIs) targeting the PD-1/PD-L1 axis prevent the interaction of both to maintain the continuous activation of T cells and enhance antitumor immune activity (134). Such drugs have shown clinical efficacy in patients who are microsatellite-instability-high (MSI-H) or mismatch repair-deficient (dMMR) but accounting for only 5% of metastatic CRC. Even certain patients may suffer primary or secondary drug resistance to immune checkpoint inhibitors (135). Moreover, the efficacy of PD-1 or PD-L1 inhibitors is limited by multiple factors including PD-1/PD-L1 expression; density of tumor-infiltrating lymphocytes, tumor mutational burden, gut microbiota, circulating cells, and molecules; and patient previous history (136). Considering these limitations, the safety, better clinical activity, and combination therapy with PD-1 or PD-L1 inhibitors, such as kinase inhibitors, chemotherapeutics, targeting agents, and other checkpoint inhibitors, are gradually gaining attention (137). Researchers found that the use of antagonists or blocking of a chemokine improves the sensitivity to anti-PD-1 treatment. For example, blocking CCR2 or CCL2 (CCR2 ligand) expression in tumor cells is particularly effective in alleviating the tumor progression and overcoming the resistance to anti-PD-1 therapy after incomplete radiofrequency ablation (138). The absence or corruption of CCL5 (CCR5 ligand) increases PD-1 and PD-L1 expression and reduces the resistance to PD-1 immunotherapy in a CRC mouse model (79). Another study also focused on the combination of anti-CCL2/5 with a PD-1 ligand inhibitor. They found a novel antibody which binds and neutralizes CCL2 and CCL5 bispecifically (BisCCL2/5i). Treatment with BisCCL2/5i and PD-L1 inhibitors simultaneously produces a powerful antitumor effect and prolongs survival in the syngeneic mouse models of liver metastasis of CRC (139). NOX-A12, an inhibitor of chemokines, is conducive to inhibiting CXCL12 and improving sensitivity to anti-PD-1 therapy by attracting T cells and NK cells to the tumor (140). In short, as detailed above, combination therapy of a chemokine antagonist with other antitumor drugs has the potential to prolong survival in patients with CRC.

## 4 Diagnosis and prognosis

Several screening strategies for CRC patients have been developed, such as high-risk factor questionnaire, fecal occult blood test, fecal immunochemical test, flexible sigmoidoscopy, colonoscopy, and computed tomography colonography (141). However, the question of early CRC diagnosis has not been solved once and for all because of poor awareness, expensive screening costs, insufficient adherence, and others. Therefore, despite a degree of success in early diagnosis and reduction of mortality, further efforts are still needed to seek comprehensive screening test which is more accurate and precise (142, 143). Given the critical role of chemokines in oncogenesis and tumor progression, the concentrations of chemokines reflect the situation and prognosis of patients with CRC to some extents.

As effective biomarkers in early diagnosis and treatment of CRC, the detection of chemokines contributes to the process of making clinical decision and improves survival. The expression of CCL5/CCR5 and CXCL7 can be used separately as an indicator in the diagnosis of CRC. When CCL5, PDGF, and EphA7 are used together, the accuracy rate of diagnosis is over 95%, whereas there is no association between CCL5 and prognosis (144). Besides, serum CXCL7 is a potential biomarker in both diagnosis and prognosis for obstructive CRC patients (145). Combination of CXCL7 with CEA, CA125, and CA19-9 may perform high sensitivity and specificity in diagnosis of CRC (146). Furthermore, the CXCL8 levels in serum are significantly higher in CRC patients with distant metastasis than those without metastasis. More importantly, compared to the classical tumor marker CEA, the serum CXCL8 and its receptor CXCR2 are more valuable on the diagnostic sensitivity, predictive value of negative results, and accuracy (147, 148). Further studies on the combination of serum level of chemokines and conventional tumor markers are badly needed to accurately detect CRC at an early stage due to the additional complexity of the actual situation in CRC patients.

Some chemokines are strongly related to tumor differentiation, tumor invasion, lymph node metastasis, distant metastasis or risk for CRC-specific mortality, such as CXCL9 (20, 149), CCL20 (150), and CCL26 (151). Besides, the expression of CXCL3, CXCL8 (152, 153), CCL5, CCL11 (121), CCL18 (154), CCR2 (155) (the receptor of CCL7), and CXCR4/7+CXCL12 (156) is closely correlated with the overall survival of CRC patients. The combination of serum CCL20 and IL-17A levels contributes to early diagnosis and prognostic predictors of CRC patients (157). High serum levels of CCL3, CCL4 (158), CCL28 (159), CXCL5 (160), CXCL8 (42, 161), and CXCL13 (92) as well as expression of CXCL16 mRNA (162) indicate an adverse prognosis in CRC patients. Since the results are derived from isolated colorectal tissue specimens or data were obtained from online databases, the real situation is probably different.

Moreover, some researchers have successfully developed a biocompatible bifunctional nano-biosensor to study the interaction between CXCR2 and chemokines which can directly detect CXCL5 in human serum samples and CRC cell model within 25 min, whereas the traditional ELISA technique needs several hours (163). In the same way, more types of chemokines and cytokines may be rapidly detected by using this biotechnology.

Studies have shown that high expression of chemokines is often related to dismal outcome, although not all. Additional research is needed to understand the complicated relationship between chemokines and clinical diagnosis and prognostic factors in CRC.

## 5 Discussion and conclusions

Chemokines serve a dual role in the tumor environment: they instigate the recruitment of immune cells as attractant and participate in activation of signaling pathways. More than that, chemokines also interact with ncRNAs to be involved in the regulation of gene expression. They are involved in tumor immune escape because of the role in tumor progression. How to use these characteristics of the tumor microenvironment and tumor cells to delay tumor progression, guide treatment, and prolong patient survival is very important.

Due to the physiological and anatomical structure of the intestinal vascular, the liver and lung are the most frequent sites for CRC metastatic and spread. Although tumors can be surgically removed and treated with adjuvant or neoadjuvant chemotherapy to delay progression and get remission, the prognosis of CRC patients is not actually optimistic. The problem in relapse and drug resistance is still a stark reality. Hence, it is hoped that timely detection and limitation of possible metastatic factors in CRC patients before metastasis can delay the progression of the disease and prolong the life of patients. Therefore, we are particularly concerned about these pre-metastasis findings. For example, elevated CXCL1 upregulated by CCRK causes the enrichment of PMN-MDSCs in the liver microenvironment and disturbs NK cell-mediated immunosurveillance, resulting in a liver pre-metastatic niche (82). In like manner, VEGF-A secreted by primary tumor cell induces primary tumor-infiltrating macrophages to produce CXCL1 which recruits MDSCs from the circulatory system into pre-metastatic liver *via* the CXCL1-CXCR2 axis, and these MDSCs promote cancer cell survival (164). Besides, the expression of CCL12, CCR2, CCR5, and CXCR2 is also elevated in the pre-metastatic liver lesions (165). Targeting CCR5 significantly inhibits CRC liver metastasis in the animal model (166). In future investigations, we might focus on researching the mechanisms of chemokine secretion and metastatic niche formation.

Another point to be considered regards drug resistance. The signaling networked system shaped by chemokines participates in chemoresistance. Combination anti-chemokine therapy and other chemotherapy drugs may be more sensitive and delay the occurrence of drug resistance.

In the present review, we outlined the function of chemokines in immune responses, cancer progression, chemoresistance, and diagnostic and prognostic value about CRC. Chemokines and other factors in the tumor constitute a complex network. Moreover, combination of chemokine antagonists and antitumor therapy appears to be an excellent strategy for prolonging survival in patients with CRC. Despite decades of study, there are still no definitive link between chemokines and tumor. Conflicting reports suggest that a deeper understanding of the exact roles of each chemokine in tumor is urgently needed.

## Author contributions

QZ, XL, and AX drafted the manuscript. ZQL, QLH, XJH, and GXX were involved in data gathering. FQT, YLD and WZ revised the manuscript. All authors read and approved the final manuscript.

## Funding

This work was supported by the National Natural Science Foundation of China (81472275), the Natural Science Foundation of Guangdong Province (2020A151501303, 2014A030313542), the Basic and Applied Basic Research Foundation of Guangdong Province Regional Joint Fund Project (The Key Project, 2020B1515120021), the Discipline Construction Project of Guangdong Medical University (4SG21276P), major projects of key platforms for colleges and universities in Guangdong Province (2020KTSCX045), and Research Fund of Guangdong Medical University (GDMUZ2020001).

## Conflict of interest

The authors declare that the research was conducted in the absence of any commercial or financial relationships that could be construed as a potential conflict of interest.

## Publisher’s note

All claims expressed in this article are solely those of the authors and do not necessarily represent those of their affiliated organizations, or those of the publisher, the editors and the reviewers. Any product that may be evaluated in this article, or claim that may be made by its manufacturer, is not guaranteed or endorsed by the publisher.
